# Differences in long-term continence rates between prostate cancer patients with extraprostatic vs. organ-confined disease undergoing robotic-assisted radical prostatectomy: An observational studys

**DOI:** 10.1016/j.clinsp.2023.100284

**Published:** 2023-09-30

**Authors:** Cristina Cano Garcia, Mike Wenzel, Florestan Koll, Agnes Zatik, Jens Köllermann, Markus Graefen, Derya Tilki, Pierre I. Karakiewicz, Luis A. Kluth, Felix K.H. Chun, Philipp Mandel, Benedikt Hoeh

**Affiliations:** a Goethe University Frankfurt, University Hospital Frankfurt, Department of Urology, Germany; b Cancer Prognostics and Health Outcomes Unit, Division of Urology, University of Montréal Health Center, Montréal, Québec, Canada; c Martini-Klinik Prostate Cancer Center, University Hospital Hamburg-Eppendorf, Hamburg, Germany; d Dr. Senckenberg Institute of Pathology, University Hospital Frankfurt, Frankfurt am Main, Germany; e Department of Urology, University Hospital Hamburg-Eppendorf, Hamburg, Germany; f Department of Urology, Koc University Hospital, Istanbul, Turkey

**Keywords:** Long-term continence, Extraprostatic, Organ-confined, Robotic-assisted radical prostatectomy, FFLU, NeuroSAFE

## Abstract

**Objectives:**

Within the tertiary-case database, the authors tested for differences in long-term continence rates (≥ 12 months) between prostate cancer patients with extraprostatic vs. organ-confined disease who underwent Robotic-Assisted Radical Prostatectomy (RARP).

**Method:**

In the institutional tertiary-care database the authors identified prostate cancer patients who underwent RARP between 01/2014 and 01/2021. The cohort was divided into two groups based on tumor extension in the final RARP specimen: patients with extraprostatic (pT3/4) vs. organ-confined (pT2) disease. Additionally, the authors conducted subgroup analyses within both the extraprostatic and organ-confined disease groups to compare continence rates before and after the implementation of the new surgical technique, which included Full Functional-Length Urethra preservation (FFLU) and Neurovascular Structure-Adjacent Frozen-Section Examination (NeuroSAFE). Multivariable logistic regression models addressing long-term continence were used.

**Results:**

Overall, the authors identified 201 study patients of whom 75 (37 %) exhibited extraprostatic and 126 (63 %) organ-confined disease. There was no significant difference in long-term continence rates between patients with extraprostatic and organ-confined disease (77 vs. 83 %; p = 0.3). Following the implementation of FFLU+ NeuroSAFE, there was an overall improvement in continence from 67 % to 89 % (Δ = 22 %; p < 0.001). No difference in the magnitude of improved continence rates between extraprostatic vs. organ-confined disease was observed (Δ = 22 % vs. Δ = 20 %). In multivariable logistic regression models, no difference between extraprostatic vs. organ-confined disease in long-term continence was observed (Odds Ratio: 0.91; p = 0.85).

**Conclusion:**

In this tertiary-based institutional study, patients with extraprostatic and organ-confined prostate cancer exhibited comparable long-term continence rates.

## Highlights

• No difference in long-term continence in extraprostatic vs. organ-confined prostate cancer.
• Even after multivariable adjustment, no difference between these two groups was identified.

## Introduction

Prostate cancer is one of the most common cancers among men in Europe.^1^ Robotic-Assisted Radical Prostatectomy (RARP) yields favorable cancer control outcomes in prostate cancer patients with clinically localized and locally advanced disease.^2^, ^3^, ^4^, ^5^, ^6^, ^7^ In recent times, the enhanced comprehension of the functional anatomy of the prostate has resulted in a heightened emphasis on functional outcomes among prostate cancer patients, particularly urinary continence, due to its significant impact on health-related quality of life.^3^^,^^8^, ^9^, ^10^, ^11^, ^12^, ^13^ Overall, there is limited data available on the long-term continence outcomes related to the tumor stage.^14^ Moreover, conflicting data from large-scale institutional databases exist regarding the impact of extraprostatic vs. organ-confined disease on continence at any given time point.^15^, ^16^, ^17^ The aim of this study was to investigate the long-term differences in continence between prostate cancer patients with extraprostatic vs. organ-confined disease who underwent RARP at the high-volume and tertiary-care hospital. Furthermore, the studied institute adopted the Full Functional-Length Urethra preservation (FFLU) and neurovascular structure-adjacent frozen-section examination (NeuroSAFE) as the new standard of care in 11/2017, leading to improved continence rates.^18^ However, it remains uncertain to what extent the implementation of this new surgical standard (FFLU+NeuroSAFE) has improved rates for both extraprostatic, as well as organ-confined disease. The authors hypothesized modest long-term continence differences between prostate cancer patients with extraprostatic vs. organ-confined disease. Additionally, the authors hypothesized that modest differences exist in the rates of continence improvement among extraprostatic vs. organ-confined diseases following the implementation of the new surgical technique. To address these hypotheses, the authors compared long-term continence rates between patients with extraprostatic vs. organ-confined disease. Subgroup analyses were conducted to assess differences in continence rates before and after implementation of FFLU+NeuroSAFE within the two groups.

## Material and methods

### Patient population

Within the prospectively collected and retrospectively analyzed European tertiary-care database of prostate cancer patients, the authors identified patients who underwent RARP between 01/2014 and 01/2021 (n = 578). Stratification was performed according to extraprostatic (pT3/4) vs. organ-confined (pT2) disease regarding tumor extension in the final RARP specimen. Therefore, patients without information about tumor stage were excluded (n=1). Additionally, the study cohort was stratified into two eras: before 11/2017 (n = 158), representing the “standard” period, and since 11/2017 (n = 419), which corresponds to the implementations of FFLU+ NeuroSAFE as a new surgical technique. Specifically, prior to 11/2017 the indication for Neurovascular Bundle Preservation (NVBP) was based on preoperative tumor information such as data derived from prostate magnetic resonance imaging, the D'Amico risk classification, as well as by using the nomograms provided by Kattan et al. and Steuber et al.^19^^,^^20^ Additionally, RARP was performed without FFLU. In 11/2017, the new standard of NeuroSAFE and FFLU was introduced in the department, including adopting FFLU combined with routine Intraoperative Frozen section analysis (IFT) guided NVBP.^21^^,^^22^ In cases where IFT revealed positive surgical margins at the site of neurovascular bundle resection, a secondary resection of the affected site was routinely performed. The FFLU technique contains the complete preservation of the entire length of the functional urethral sphincter by identifying and dissecting the striated and smooth muscle part of the urethral sphincter inside the prostate apex until the colliculus.^13^^,^^21^

In the current study, only patients with available long-term continence data (≥ 12 months) were included (n=212). Exclusion criteria consisted of patients with neoadjuvant Androgen Deprivation Therapy (ADT, n=6) or/and clinical suspicion of metastases (n=5) to give a final cohort for analysis of 201 study patients (before 11/2017 n = 70 and FFLU+NeuroSAFE n = 131). Ethical approval was obtained from the institutional review boards of the University Cancer Center Frankfurt and the Ethical Committee at the University Hospital Frankfurt (SUG-1-2018_12021, Amendment 3 2021), and written informed consent was obtained from all patients. The reporting of this observational study followed the STROBE guidelines (= STrengthening the Reporting of OBservational studies in Epidemiology).

### Outcome measures

Long-term continence information was collected by voluntary standardized self-reported questionnaires, as previously described.^3^^,^^23^. For the present analysis, the first event of continence at least 12 months after RARP was considered. Long-term urinary continence was defined as the absence or usage of only one safety pad within 24 hours at least 12 months after RARP. On the other hand, if a patient required the use of more than one safety pad, they were categorized as experiencing incontinence. Differences in long-term continence rates between patients with extraprostatic vs. organ-confined disease were quantified. Subsequently, the authors tested for continence rate differences between extraprostatic and organ-confined disease within the standard surgical technique vs. the newly implemented FFLU+NeuroSAFE.

### Statistical analyses

Descriptive statistics included frequencies and proportions for categorical variables. Medians and Interquartile Ranges (IQR) were reported for continuously coded variables. Kruskal-Wallis rank sum test and Pearson's Chi-Squared test or Fisher's exact test tested for statistically significant differences in medians and proportions, respectively. Finally, multivariable logistic regression analyses tested for differences between prostate cancer patients with extraprostatic vs. organ-confined disease in analyses addressing long-term continence. Covariables consisted of baseline age, Body Mass Index (BMI), and prostate volume as continuous variables, as well as surgical technique (standard vs. FFLU+NeuroSAFE), nerve sparing (complete bilateral vs. bilateral with one side partial vs. unilateral vs. no nerve sparing), surgical margin (R0 vs. R1 vs. RX) and pN-stage (pN0 vs. pN1 vs. pNX) as categorical variables. The significance level was set at p < 0.05. For all statistical analyses, R Software Environment for Statistical Computing and Graphics (R version 4.1.3, R Foundation for Statical Computing, Vienna Austria) was used.^24^

## Ethics

The study was conducted according to the guidelines of the Declaration of Helsinki and approved by the institutional review boards of the University Cancer Centre Frankfurt and the Ethical Committee at the University Hospital Frankfurt.

## Results

### Descriptive characteristics

Overall, the authors identified 201 study patients of whom 75 (37 %) exhibited extraprostatic and 126 (63 %) organ-confined disease. The authors observed important differences in patients with extraprostatic vs. organ-confined disease. Specifically, patients with extraprostatic disease exhibited higher rates of Gleason grade group V in biopsy (11 % vs. 1 %; p < 0.001) and RARP specimen (20 % vs. 3 %, p < 0.001), more frequent D'Amico high-risk group (35 % vs. 10 %; p < 0.01), as well as higher rates of unilateral and no nerve sparing (bilateral 45 % vs. 71 %, bilateral with one side partial 14 % vs. 11 %, unilateral 20 % vs. 8 % and no nerve sparing 21 % vs. 10 %, p = 0.003), pN1 (12 % vs. 0 %, p < 0.001) and positive surgical margins (R1 53 % vs. 10 %; p < 0.001), relative to their organ-confined counterparts. Conversely, no differences between patients with extraprostatic vs. organ-confined disease were observed according to age at diagnosis, BMI, prostate volume, and performed FFLU+NeuroSAFE (Table 1).

**Table 1:** Descriptive characteristics of 201 prostate cancer patients undergoing Robotic-Assisted Radical Prostatectomy (RARP) and available long-term continence information between 01/2014 and 01/2021, stratified according to extraprostatic vs. organ-confined disease.

Characteristic	Overall	Extraprostatic^a^	Organ-confined^a^	p-value^b^
	n = 201 (100 %)	n = 75 (37 %)	n = 126 (63 %)	
**Age at diagnosis (years)**	66 (61, 70)	67 (63, 70)	66 (60, 70)	0.6
**PSA in mg/mL**	7.0 (5.2, 10.0)	9.0 (6.3, 12.0)	6.7 (5.0, 9.4)	<0.001
**BMI in kg/m^2^**	26.4 (24.5, 28.7)	27.1 (24.7, 29.9)	26.0 (24.2, 28.4)	0.11
**Prostate volume in cm^3^**	39 (30, 52)	35 (30, 46)	40 (30, 59)	0.036
**Gleason grade group Biopsy-specimen**				<0.001
I	51 (25 %)	10 (13 %)	41 (33 %)	
II	92 (46 %)	35 (47 %)	57 (45 %)	
III	32 (16 %)	15 (20 %)	17 (13 %)	
IV	17 (8.5 %)	7 (9 %)	10 (8 %)	
V	9 (4.5 %)	8 (11 %)	1 (1 %)	
**D'Amico risk groups**				<0.001
Low	35 (18 %)	6 (8 %)	36 (24 %)	
Intermediate	125 (63 %)	43 (57 %)	97 (66 %)	
High	39 (20 %)	26 (35 %)	15 (10 %)	
**Nerve sparing**				0.003
Complete bilateral	119 (61 %)	32 (45 %)	87 (71 %)	
Bilateral with one side partial	24 (12 %)	10 (14 %)	14 (11 %)	
Unilateral	24 (12 %)	14 (20 %)	10 (8 %)	
No	27 (14 %)	15 (21 %)	12 (10 %)	
**FFLU+NeuroSAFE**	131 (65 %)	83 (66 %)	47 (64 %)	0.8
**Gleason grade group RP-specimen**				<0.001
I	40 (20 %)	5 (7 %)	35 (28 %)	
II	103 (51 %)	36 (48 %)	67 (53 %)	
III	31 (15 %)	15 (20 %)	16 (13 %)	
IV	9 (4 %)	4 (5 %)	5 (4 %)	
V	18 (10 %)	15 (20 %)	3 (3 %)	
**pN-stage**				<0.001
pN0	179 (89 %)	62 (83 %)	117 (93 %)	
pN1	9 (4 %)	9 (12 %)	0 (0 %)	
pNx	13 (7 %)	4 (5 %)	9 (7 %)	
**Surgical margins**				<0.001
R0	145 (72 %)	33 (44 %)	112 (89 %)	
R1	53 (26 %)	40 (53 %)	13 (10 %)	
RX	3 (2 %)	2 (3 %)	1 (1 %)	

a Median (IQR, Interquartile Range); n ( %)

b Kruskal-Wallis rank sum test; Pearson's Chi-Square test; Fisher's exact test. PSA, Prostate Specific Antigen; FFLU, Full Functional-Length Urethral Sphincter Preservation; NeuroSAFE, Neurovascular Structure-Adjacent Frozen-Section Examination.

### Long-term continence rates

**Figure 1:**
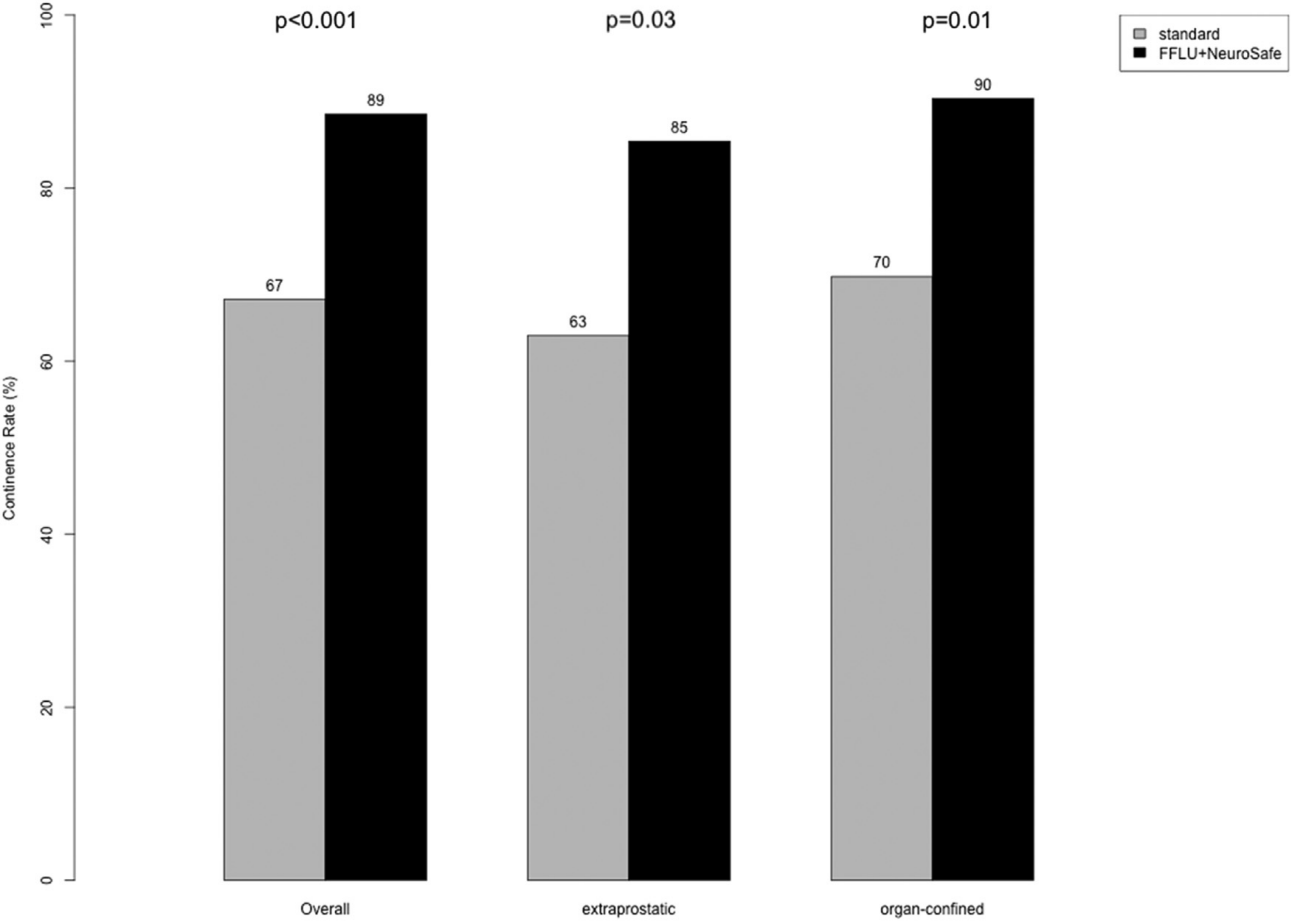
Comparison of long-term (≥ 12 months) continence rate of patients undergoing Roboter-Assisted Radical Prostatectomy stratified according to extraprostatic vs. organ-confined disease between eras: before 11/2017 (= Standard) and since 11/2017 (= Implementation of Full Functional-Length Urethra preservation [FFLU] and Neurovascular Structure-Adjacent Frozen-Section Examination [NeuroSAFE]).

There was no significant difference in long-term continence rates observed between prostate cancer patients with extraprostatic vs. organ-confined disease (77 % vs. 83 %; p = 0.3, Table 2). However, the overall continence improved in both extraprostatic and organ-confined prostate cancer patients after the implementation of FFLU+NeuroSAFE. Specifically, for patients with extraprostatic disease, there was a significant improvement in continence by 22 % (from 63 % to 85 %, p = 0.03). Similarly, patients with organ-confined disease also exhibited significant improvement in continence by 20 % (from 70 % vs. 90 %, p = 0.01; Fig. 1).

**Table 2:** Long-term continence rate of patients undergoing Robotic-Assisted Radical Prostatectomy (RARP) between 01/2014 and 01/2021, stratified according to extraprostatic vs. organ-confined disease.

Characteristic	Overall (n = 201)	Extraprostatic^a^, n = 75 (37 %)	Organ-confined^a^, n = 126 (63 %)	p-value^b^
Long-term continence				0.3
Yes	163 (81 %)	58 (77 %)	105 (83 %)	
No	38 (19 %)	17 (23 %)	21 (17 %)	

a n ( %).

b Kruskal-Wallis rank sum test; Pearson's Chi-Square test; Fisher's exact test.

### Multivariable logistic regression models

In the multivariable logistic regression models, no significant difference in long-term continence was observed between prostate cancer patients with extraprostatic and organ-confined disease (Odds Ratio [OR = 0.91; 95 % Confidence Interval [95 % CI 0.34‒2.50, p = 0.85; Table 3).

**Table 3:** Multivariable^a^ logistic regression models predicting long-term (≥12 months) urinary continence^b^ in 201 patients treated with Robotic-Assisted Radical Prostatectomy (RARP).

	Multivariable		
	Odds ratio	95 % confidence interval	p-value
**Extraprostatic disease**^b^			
No	Reference	–	–
Yes	0.91	0.34 – 2.50	0.85

a Adjusted for surgical technique (FFLU+NeuroSAFE vs. standard), nerve sparing status, pN stage, as well as age, BMI and prostate volume as continuous variables.

b Urinary continence was defined by usage of no or one safety pad within 24h. FFLU, Full Functional-Length Urethral Sphincter Preservation; NeuroSAFE, Neurovascular structure-adjacent frozen-section examination; pN stage, Pathological lymph node stage; BMI, Body Mass index.

## Discussion

In the current study, the authors investigated long-term continence outcomes in prostate cancer patients with extraprostatic vs. organ-confined disease who underwent RARP. Moreover, the authors examined the differences in continence rates before and after the implementation of FFLU+NeuroSAFE within both groups. The authors hypothesized modest long-term continence differences between prostate cancer patients with extraprostatic vs. organ-confined disease. Additionally, the authors hypothesized that modest differences exist in the rates of continence improvement among extraprostatic vs. organ-confined diseases following the implementation of the new surgical technique. The authors tested these hypotheses within the institutional tertiary-care database and made several important observations.

First, the authors observed important differences in patient, tumor, and surgical characteristics between prostate cancer patients with extraprostatic vs. organ-confined disease. Specifically, patients with extraprostatic disease showed a higher frequency of the D'Amico high-risk group (35 % vs. 10 %; p < 0.01), as well as higher rates of pN1 (12 % vs. 0 %, p < 0.001) and positive surgical margins (R1 53 % vs. 10 %; p < 0.001), relative to their organ-confined counterparts. The rate of positive surgical margins in extraprostatic disease patients (53 %) in the current study is close to the overall rate reported by van der Slot et al. (48 %).^25^ The more unfavorable rates of positive surgical margins could also be associated with the fact that the present institution serves as both a tertiary care hospital and a teaching hospital, resulting in a diverse range of surgical expertise. However, it is worth noting that the group previously observed decreased rates of positive surgical margins following the implementation of FFLU+NeuroSAFE, even among patients with pT3 (from 47.1 % to 29.4 %).^22^ Moreover, in the current study, patients with extraprostatic disease had lower rates of nerve sparing compared to patients with organ-confined disease (bilateral 45 % vs. 71 %, bilateral with one side partial 14 % vs. 11 %, unilateral 20 % vs. 8 % and no nerve sparing 21 % vs. 10 %, p = 0.003). Underlying differences in patient, tumor, and surgical characteristics may influence functional outcomes such as urinary continence.^26^ For example, in their Meta-Analysis Nguyen et al. reported that nerve sparing was associated with better urinary continence in prostate cancer who underwent radical prostatectomy. Specifically, 12 months after surgery the risk of urinary incontinence was lower when bilateral nerve sparing was performed.^27^ Consequently, to account for these underlying differences, the authors performed multivariable adjustments in the current analyses focusing on long-term continence.

Second, the authors recorded no significant difference in long-term continence rates between patients with extraprostatic and organ-confined disease within the entire cohort (77 % vs. 83 %, p = 0.3). Moreover, even after adjusting for patient, tumor, and surgical characteristics using multivariable logistic regression models, the authors still observed no long-term continence differences between both tumor stage groups (OR = 0.91; 95 % CI 0.34‒2.50, p = 0.85). The data regarding the impact of extraprostatic disease on long-term continence (at least 12 months) appear to be heterogeneous.^15^, ^16^, ^17^ The results of the current study align with previous analyses where no significant differences in long-term continence rates between extraprostatic vs. organ confined were observed.^15^^,^^17^^,^^18^ However, it is worth noting that Grabbert et al. identified tumor stage as an independent predictor for continence in their multivariable analysis.^16^

Third, the authors observed significant differences in long-term continence rates before and after the implementation of a new surgical approach (FFLU+NeuroSAFE). Specifically, the overall long-term continence rate increased from 67 % to 89 % (Δ = 22 %; p < 0.001). This improvement was consistent for both patients with extraprostatic disease (63 % vs. 85 %; Δ = 22 %; p = 0.03) and patients with organ-confined disease (70 % vs. 90 %; Δ = 20 %; p = 0.01). The current results align with data reported by Schlomm et al. where significantly improved early continence rates were observed after the implementation of the FFLU surgical technique.^21^ In consequence, the findings of the current study indicate that the implementation of FFLU+NeuroSAFE had a substantial positive impact on the long-term continence rates for both patients with extraprostatic and organ-confined patients.

Taken together, the authors made important observations regarding the association of tumor stage and long-term continence in RARP patients. Specifically, the authors observed no differences in long-term continence rates between prostate cancer patients with extraprostatic vs. organ-confined disease undergoing RARP, even after adjusting for patient, tumor, and surgical characteristics multivariable analyses. These observations hold considerable importance in patient education and should be discussed during informed consent discussions. Furthermore, the present results suggest that a surgical approach in patients with extraprostatic disease can still lead to favorable continence rates.

The current study has several limitations that need to be acknowledged. First and foremost, the retrospective design and the limited sample size, specifically the limited availability of long-term continence data of patients undergoing RARP in the present institute, inherently contribute to potential biases and may limit the generalizability of the findings. Second, information on long-term continence derived from voluntary, self-questionnaire reports. As a result, the current study may be subject to selection and non-response bias. Third, although the authors adjusted for available patient and tumor characteristics in multivariable models, it is essential to recognize that certain potentially significant factors, such as comorbidities, could not be accounted for, which may have influenced continence outcomes. Fourth, prostate cancer patients undergoing RARP receive in-patient professional pelvic floor training after surgery. Additionally, further pelvic floor training in outpatient settings is highly recommended. However, variations in the performance and duration of this professional pelvic-floor training among patients in the present study may have impacted the continence rates observed. Moreover, the Coronavirus Disease 19 (COVID-19) pandemic may have influenced the availability of professional pelvic-floor training, potentially affecting the results. In their randomized controlled trial, Overgård et al. reported lower rates of urinary incontinence after 12 months in patients receiving instructed pelvic floor training by a physiotherapist compared to patients performing the training on their own.^28^ Finally, since the study includes prostate cancer patients who underwent RARP performed by different surgeons (n = 9) with varying levels of experience, the possibility of differences in surgical expertise influencing the outcomes should be acknowledged.

## Conclusion

In this tertiary-based institutional study, patients with extraprostatic and organ-confined prostate cancer exhibited comparable long-term continence rates.

## Funding

The research was conducted in the absence of any commercial or financial relationship that could be construed as a potential conflict of interest.

## CRediT authorship contribution statement

**Cristina Cano Garcia:** Conceptualization, Data curation, Formal analysis, Investigation, Methodology, Writing – original draft. **Mike Wenzel:** Data curation, Formal analysis, Investigation, Writing – review & editing. **Florestan Koll:** Data curation, Investigation, Writing – review & editing. **Agnes Zatik:** Investigation, Writing – review & editing. **Jens Köllermann:** Data curation, Writing – review & editing. **Markus Graefen:** Writing – review & editing. **Derya Tilki:** Writing – review & editing. **Pierre I. Karakiewicz:** Supervision, Writing – review & editing. **Luis A. Kluth:** Supervision, Writing – review & editing. **Felix K.H. Chun:** Supervision, Writing – review & editing. **Philipp Mandel:** Conceptualization, Methodology, Supervision, Writing – review & editing. **Benedikt Hoeh:** Conceptualization, Data curation, Formal analysis, Investigation, Methodology, Supervision, Writing – review & editing.

## Declaration of Competing Interest

The authors declare no conflicts of interest.
